# Anterior Closing Wedge Osteotomy for Failed Anterior Cruciate Ligament Reconstruction: State of the Art

**DOI:** 10.5435/JAAOSGlobal-D-22-00044

**Published:** 2022-09-16

**Authors:** Anshu Shekhar, Sachin Tapasvi, Ronald van Heerwaarden

**Affiliations:** From the Sushrut OrthoPlastic Clinic, Raipur, India (Dr. Shekhar); The Orthopaedic Speciality Clinic, Pune, India (Dr. Tapasvi); Centre for Deformity Correction and Joint Preserving Surgery, Kliniek ViaSana, Mill, the Netherlands (Dr. van Heerwaarden); International Knee and Joint Centre, Abu Dhabi, United Arab Emirates (Dr. van Heerwaarden); and London Osteotomy Centre, Harley Street Specialist Hospital, London, United Kingdom (Dr. van Heerwaarden).

## Abstract

The sagittal anatomy of the proximal tibia has a bearing on the forces exerted on the cruciate ligaments. A high posterior tibial slope is now a well-known risk factor causing failure of anterior cruciate ligament (ACL) reconstructions. The posterior slope can be calculated on short or full-length radiographs, MRI scans, or three-dimensional CT scans. Reducing the slope surgically by a sagittal tibial osteotomy is biomechanically protective for the ACL graft. An anterior closing wedge osteotomy may be contemplated when the lateral tibial slope is greater than 12°, in the setting of ACL reconstruction failure(s). Careful surgical planning to calculate the correction, taking into account knee hyperextension and patella height, is critical to avoid complications. It can be done above, at, or below the tibial tuberosity level. A transtuberosity correction can be done with or without a tibial tubercle osteotomy. This complex surgery can be conducted safely by meticulous execution to protect the posterior hinge and neurovascular structures and achieving stable fixation with staples. The limited literature available justifies the usage of anterior closing wedge osteotomy in appropriately selected patients.

Anterior cruciate ligament (ACL) tears are common injuries after contact and noncontact injuries with multifactorial pathoetiology. The anatomic factors implicated in the causation of ACL tears include a narrow stenotic intercondylar notch, shallow concavity of the medial tibial plateau, increased posterior tibial slope (PTS), and increased curvature of the femoral and tibial condyles.^1-3^ Two of these factors—a narrow notch and an increased PTS— have drawn the most attention, probably because they can be modified surgically. The contribution of a high PTS in noncontact ACL injury has been well known and reported since past 15 years.^2,4-6^ This association between a high PTS and ACL reconstruction (ACLR) failure is very pertinent in the clinical setting. The rates of ipsilateral autograft ACL retear in a patient with a high PTS ranges from 2.8% to 15%, depending on the age, activity, and return to sports.^7,8^ Both medial and lateral PTSs are independent risk factors for ACLR retear, irrespective of whether the tunnels are anatomic or nonanatomic.^9^ In adolescents, a high PTS is associated with ACLR retears in the short and long term and is more pronounced.^10-15^

The understanding of the morphology of the tibial slope has also been consistently evolving. The osseous slope consists of the medial and lateral tibial plateaus, and their variations can be wide-ranging. The medial slope is usually steeper than the lateral slope.^16,17^ One also needs to consider the soft-tissue components, such as the cartilage and meniscus, in the assessment of the slope. The cartilages and menisci are thicker posteriorly than anteriorly, which causes a reduction in the “soft-tissue slope” vis-à-vis the bony slope. The effect of soft tissues in making the overall slope more horizontal is greater in the lateral compartment and more so when the bony slope is steeper.^18,19^ A lateral meniscus slope of >4° is correlated with increased anterior translation after ACLR. Batty et al. reported that the biomechanical effect of a high tibial slope is accentuated by a medial meniscus injury in its posterior third. Such a tear has an odds ratio of 2.55 for a grade 3 pivot shift test, irrespective of the PTS being greater or lesser than 9°. However, when the meniscus was intact, only a PTS of >9° was associated with a high-grade pivot.^20^ This study aims to review the biomechanical effect of high PTS on ACL, techniques of measuring the PTS, indications and techniques of anterior closing wedge osteotomy (ACWO), and current literature on the results of an ACWO.

## Biomechanical Effect of Tibial Slope on Anterior Cruciate Ligament

**Figure 1:**
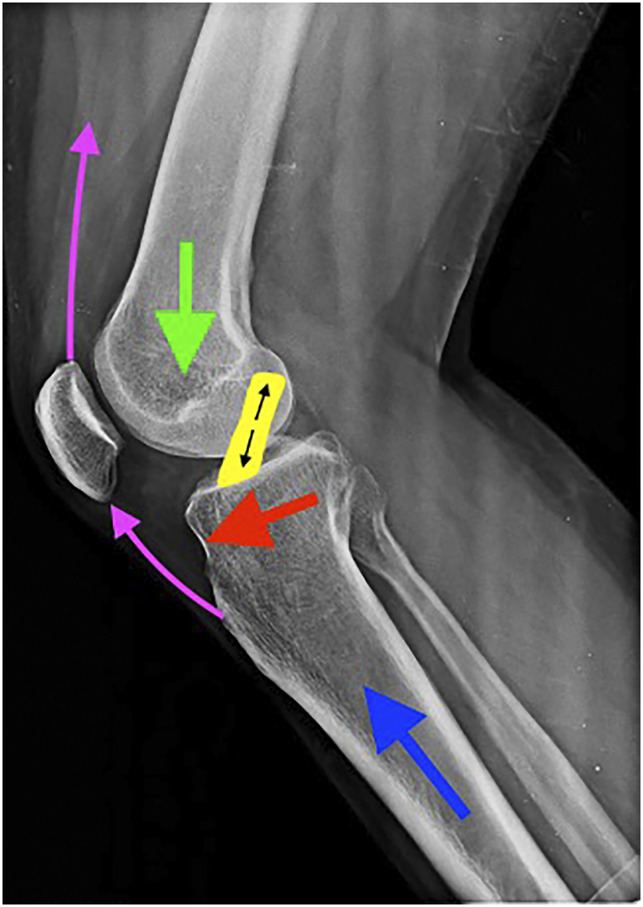
Radiograph showing that biomechanical forces in play in the knee are the joint loading directed distally (green arrow), ground reaction force directed proximally (blue arrow), superior pull of the extensor apparatus (pink arrows), and anterior translatory force (red arrow), which is resisted by the tension of the intact anterior cruciate ligament (yellow band).

In vivo studies have sought to understand the effect of PTS on knee kinematics, specifically in relation to ACL biomechanics (Figure 1). A high PTS causes greater static anterior translation of the lateral compartment in an ACL injured knee.^6^ The lateral PTS is higher in those with a torn ACL compared with control subjects with an intact ACL.^5,21^ Beynnon et al^22^ reported the increased risk of a noncontact ACL tear with a high lateral slope in female athletes only. Giffin et al demonstrated that a 5-mm ACWO to increase the PTS resulted in an anterior shift of the resting position of the tibia in relation to the femur by a mean of 3.6 ± 1.4 mm. They postulated that reducing the slope would similarly lead to a posterior translational effect on the tibia.^23^ McLean et al conducted a simulated jump-landing task on 11 cadaveric lower limbs to understand impact-induced anterior tibial translation (ATT) and resultant strain on the ACL. They found notable correlation between PTS with peak ATT (*r* = 0.75) and peak anteromedial bundle ACL strain (*r* = 0.76).^24^ Another cadaver experiment by Bernhardson et al demonstrated a highly positive linear relationship between tibial slope and force in an ACL graft, when the knee is axially loaded. This relationship was found to exist for flexion angles of 0°, 15°, 30°, 45°, and 60°, but the graft force was markedly higher at 0°.^25^ Thus, PTS has a bearing on the ACL tension, both in the static position and the loaded knee.

Yamaguchi et al studied the effects of a 10° anterior closing wedge osteotomy on ACLs in human cadaveric knees using a robotic setup. The osteotomy reduced ACL force and ATT on application of tibiofemoral compression, either alone or in combination with an anterior force or valgus moment.^26^ Imhoff et al also demonstrated a reduction of ATT on axial loading after a 10° anterior closing wedge osteotomy. The ACLR graft force on a quadrupled hamstring graft reduced by mean 17% on applying 200 N axial load and by mean 33.1% on applying 400 N axial load.^27^ These in vivo experiments provide rationale for the protective effect of an ACWO on the ACL graft.

## Measurement of the Tibial Slope

There are several methods which have been described to measure PTS. PTS can be measured on sagittal radiographs—with short radiographs or full-length tibial views, MRI scans, or three-dimensional (3D) CT scans. Each technique has its merits and limitations. The available literature on high PTS and ACL injury uses each of these modalities, but the values obtained are not interchangeable, which makes comparison difficult. The values of PTS measured on MRI are lower than those measured on radiographs.^28^ Moreover, there is no consensus on whether to use the anatomic axis, the mechanical axis, or the posterior cortical line as the reference, and there can be variations because of this as well.^29^ A short radiograph is most widely used but does not consider soft tissues, is prone to errors because of rotation, and cannot accurately measure the medial and lateral slopes separately. An MRI scan overcomes all these barriers but is technically difficult and has decreased acceptability.^28^ A 3D CT scan can accurately measure the bony slope on either plateau but exposes the patient to radiation and entails additional cost. Unfortunately, there is no standardization of either the tool or the technique used for measuring or reporting PTS in current literature. Some of the most commonly used methods using each modality are described below.

### Short Lateral Radiograph

**Figure 2:**
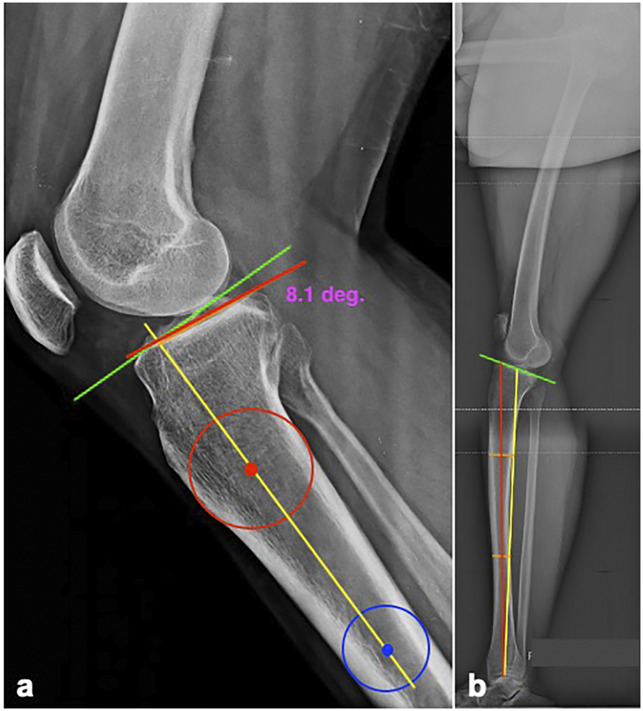
**A**, Measurement of the slope on a short lateral radiograph: The centers of circles touching the anterior and posterior tibial cortices at 5 cm. (red dot) and 15 cm. (blue dot) distal to the joint line are marked and joined to obtain the anatomic axis of the tibia (yellow line), which is then extended proximally to the joint level. A second line is drawn perpendicular to this line centering on the tibial articular surface (green line), and a third line is drawn along the lateral tibial plateau (orange line). The angle subtended between the second and third lines is the posterior tibial slope, 8.1° in this case. **B**, Measurement of the slope on a full-length lateral radiograph: The anatomic axis of the tibia (yellow line) is drawn by joining the centers of the tibial plafond and tibial plateau in the lateral view. The mechanical axis is drawn by joining the center points between the anterior and posterior cortices of the tibial diaphysis at two points (orange lines) and extending this line proximally to the tibial plateau (red line). A tangent along the tibial articular surface (green line) yields the posterior tibial slope.

The radiograph must have at least 20 cm. of the tibia visible, and the femoral condyles must be perfectly overlapped to prevent rotation. It is usually difficult to identify, but the lateral tibial slope should preferably be measured when possible. There are several techniques but the one described by Utzschneider is easily reproducible^30^ (Figure 2, A).

### Full-length Lateral Monopedal Stance Radiograph

This radiograph allows PTS to be measured along either the mechanical axis or the anatomic axis of the tibia in the sagittal plane^31^ (Figure 2, B). It has been demonstrated that when the anatomic axis is used as a reference, the values obtained on short and full-length radiographs are similar.^29^ Nonetheless, the distinct advantage of a weight-bearing monopedal stance lateral radiograph is that it allows an assessment of knee hyperextension and static ATT in the loaded knee.

### MRI Scan

**Figure 3:**
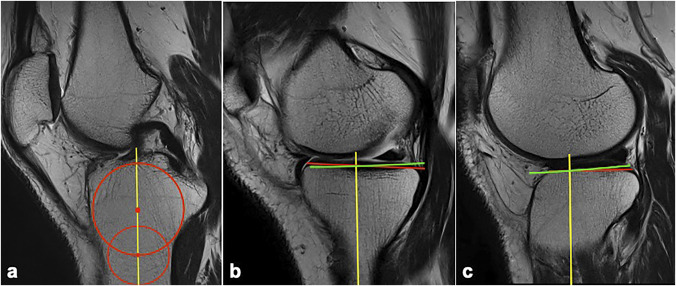
**A**, Image of the central sagittal slice of the MRI scan, which shows the tibial posterior cruciate ligament attachment and the intercondylar eminence. Two circles were drawn (red)—one cranial, touching the anterior and posterior cortices and the joint surface, and one caudal, touching the anterior and posterior cortices. The centers of these circles are joined and extended proximally to obtain the longitudinal axis (LA) (yellow line). **B**, Image showing the medial posterior tibial slope (PTS) is calculated in the central mediolateral section of the medial plateau. The LA is projected as previously drawn (yellow line). A line perpendicular to the LA is drawn (green line); a tangent is drawn along the articular surface of the cartilage (red line); and the angle between these two lines is the medial PTS. **C**, Image showing the lateral PTS calculated in the central mediolateral section of the lateral plateau. The LA is projected as previously determined (yellow line). A line perpendicular to the LA is drawn (green line); a tangent is drawn along the articular surface of the cartilage (red line); and the angle between these two lines is the lateral PTS.

The technique described by Hudek et al^32^ can be conducted without restriction of the length of the tibia exposed on the scan. It has also been validated as having the least interobserver and intraobserver variability compared with other MRI-based techniques.^33^ Sagittal T2-weighted sections are selected at the level of tibial posterior cruciate ligament (PCL) attachment for drawing the longitudinal axis of the tibia and at the midplateau levels for measuring the medial and lateral PTSs separately (Figure 3).

### Three-dimensional CT Scan

**Figure 4:**
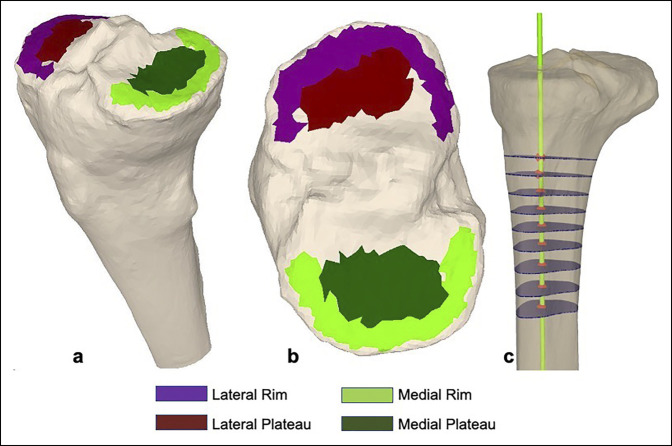
Illustrations showing four areas of the tibial articular surface brushed medially and laterally in the three-dimensional reconstruction seen superomedially (**A**) and from the top (**B**). **C**, The anatomic axis (green line) is determined by joining the centers of the tibial sections at several levels starting 15 cm distal to the articular surface. The angles between the anatomic axis and previously determined four sagittal planes yield the posterior tibial slopes. (Adapted and modified from Hoch et al.^35^)

The greatest advantage of a 3D CT scan over radiography is elimination of errors due to the rotational profile of the tibia and accurate analysis by referencing the anatomic axis.^34^ Moreover, it allows independent assessment of both medial and lateral plateaus and the peripheral rim with a high degree of reliability and accuracy^35^ (Figure 4).

## When Should Slope Reduction Be Considered?

It is important to know what a “normal” PTS is, before one considers the slope in an ACL injured knee to be high. There is a wide racial and gender variation in the values reported in healthy individuals as summarized in Table 1.^16,36-38^ A level III meta-analysis by Wang et al^39^ found that increased medial and lateral slopes are risk factors for ACL rupture only for male individuals. Hence, one needs to be aware of the prevalent mean PTS values in his/her region of practice to better decide what constitutes an “abnormal” slope.

**Table 1:** Reported Variation in Posterior Tibial Slope

Study	Geographic Location	Method of Assessment	Medial/Lateral Variation	Ethnic Variation	Sex Variation
Weinberg et al^16^	United States	Radiographs of osteology specimens	Mean mPTS = 6.9° ± 3.7° Mean lPTS = 4.7° ± 3.6°	mPTS and lPTS of Blacks > Whites	mPTS and lPTS greater in female individuals
Pangaud et al^36^	Europe	3D CT scans of healthy adults	Mean gPTS = 6.3° Mean mPTS = 6.2° Mean lPTS = 5.3°	gPTS of Asians > Whites	gPTS and lPTS smaller in female individuals
Aljuhani et al^37^	Saudi Arabia	MRI scans of healthy adults	Mean mPTS = 6.1° ± 3.1° Mean lPTS = 4.4° ± 3.1°	Only Arabs	No notable gender dimorphism
Zhang et al^38^	China	3D CT scans of healthy adults	Mean mPTS = 8.8° ± 2.8° Mean lPTS = 8.4° ± 2.7°	Only Chinese	mPTS and lPTS greater in female individuals but not statistically significant

3D = three-dimensional, gPTS = global posterior tibial slope, lPTS = lateral posterior tibial slope, mPTS = medial posterior tibial slope

The threshold of what constitutes a high PTS to merit an intervention is still debatable, considering the variations of a mean normal PTS and myriad measurement methods used.^40^ The seminal work of Webb et al was a longitudinal study of 181 ACLRs to assess the role of radiological PTS on additional ACL failure. They calculated the medial PTS on short lateral radiographs using OsiRix software, with the tibial anatomic axis as a reference. After 15 years of follow-up, they found five times higher odds of additional ACLR failure when the PTS was ≥12°, with an incidence of 59%.^10^ The same cohort was assessed at 20 years, and it was found that a PTS of ≥12° is the strongest predictor of additional ACL failure. This risk was exaggerated in adolescents, in whom the graft survival was only 22%, and the hazard increased by 11 compared with adults with a <12° slope.^11^ The mean PTS of those patients who sustained more than three ACLR failures was 12° in this cohort.^41^ In a retrospective series of 64 patients who underwent a revision ACLR, Lee found that the odds ratio of ACL graft rerupture was 4.52, when the PTS was ≥12°.^42^ In a series^36^ of 378 3D CT scans of healthy individuals of both sexes and varied ethnicities, Pangaud et al found a slope of >12° in ≦3% of all scans (1% global PTS, 3% medial PTS, and 2% lateral PTS). This does indicate that a radiologic PTS of >12° is indeed pathologic and it is reasonable to “draw a line” at this value when contemplating to reduce the slope.

Other factors to take into consideration when planning ACWO are as follows: (1) the amount of hyperextension found during clinical examination of the knee because extension of the knee increases after an ACWO. The amount of hyperextension produced after the correction can be predicted using the preoperative heel-raise height and lower leg length. A 1-cm. heel height corresponds to 1° hyperextension, if the leg segment is up to 58 cm. in length.^44^ (2) Patellar height depending on the level of wedge removal because the patella height may change in an ACWO, that is, a large ACWO above the tibial tuberosity level may cause a patella alta. (3) Integrity of the PCL since reduction of the PTS, especially when performed to less than normal values causes high stresses on the PCL (4) integrity of the menisci, as anterior translation of the tibia in the presence of an ACL tear is also influenced by avulsion of meniscal roots and meniscus tears and hence, maximum effort should be undertaken and restore meniscal anatomy in case of additional meniscal pathology.^18-20^

Thus, the absolute contraindications for conducting an ACWO are (1) knee hyperextension >10° because the patient will have difficulty to rebalance his knee afterward and because additional soft-tissue procedures to tighten the posterior capsule seem to be insufficient to withstand hyperextension beyond 10°, (2) patella alta (for a supratuberosity osteotomy), (3) PCL deficiency, and (4) grade IV (Kellgren-Lawrence) tibiofemoral osteoarthritis. Relative contraindications as for any osteotomy are a body mass index of >30 kg/m^2^ and a smoking history of 20 cigarettes per day.^45^

## Methods of Anterior Closing Wedge Osteotomy

**Figure 5:**
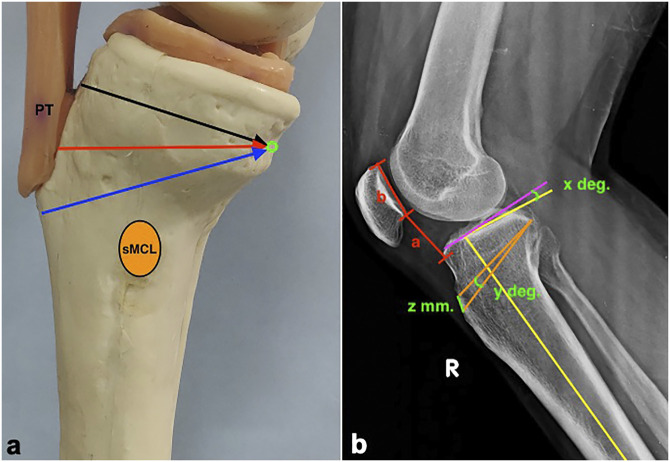
**A**, Photograph of the levels of the slope-reducing osteotomy—supratuberosity (black arrow), transtuberosity (red arrow), and infratuberosity (blue arrow)— and the hinge point on the posterior cortex at the lower half of posterior cruciate ligament insertion (green circle). **B**, Radiograph demonstrating planning for the osteotomy. The posterior slope (x°) is calculated by measuring the angle between the perpendicular to the mechanical axis (pink line) and a tangent to the joint surface (yellow line). This angle of correction (y°) is subtended on the radiograph centering at the hinge point and extending the lines to the anterior cortex. The distance (z, millimeters) between the lines ending at the anterior cortex is the height of the wedge to be removed in a calibrated radiograph. The patella height is calculated by measuring the Caton-Deschamps ratio, which is obtained by dividing the length of a by b (a/b). PT = patella tendon, sMCL = superficial medial collateral ligament.

ACWO can be conducted at three levels of tibial tuberosity—proximal to it, at the level, or distal to it (Figure 5, A). Each of these techniques has its advantages and disadvantages (Table 2).

**Table 2:** Advantages and Disadvantages of ACWO Techniques

Technique	Advantages	Disadvantages
Supratuberosity	No TTO required Better healing in pure cancellous bone Can allow usage of a BPTB graft for simultaneous revision ACL reconstruction	Only a short oblique ACL tibial tunnel is possible Potential to cause patella alta Potential to cause injury to the patella tendon Small proximal segment can compromise ACWO fixation
Transtuberosity	TTO provides safe exposure When reinserting the tibial tuberosity at its original proximal position, preoperative patella height will be preserved The tibial tuberosity can be used as a biological plate fixation^46^	Usually requires a TTO Postoperative mobilization is slower if a TTO is conducted to protect TTO bone healing
Infratuberosity	No TTO required Allows plenty of space for ACL tibial drilling Does not compromise patellofemoral mechanics	Metadiaphyseal bone has lesser healing potential than epimetaphyseal bone

ACL = anterior cruciate ligament, ACWO = anterior closing wedge osteotomy, BPTB = bone–patella tendon–bone, TTO = tibial tubercle osteotomy

## Planning of Anterior Closing Wedge Osteotomy

Reducing the PTS to approximately 6° to 8° has been recommended by some authors.^46,47^ This would bring it within the range of normal mean values in most populations. The hinge point for the closing wedge osteotomy is chosen near the posterior tibial cortex and not too close to the articular surface to prevent an intra-articular hinge fracture. When positioned within the lower half of the PCL insertion, this broad and strong tendinous structure reinforces the hinge when deformed during closing. A “rule of thumb” system to convert degrees of correction to millimeters of closing wedge has been proposed by several authors.^47-49^ However, a more accurate method is to plan the osteotomy on calibrated radiographs (Figure 5, B). The steps in this exercise are as follows:

(1) Measure the lateral slope (x°) as previously described in Figure 2.
(2) Measure the degree of correction required to reduce the slope to 6° to 8° (x° − y° = 6°)
(3) Plot y° on the radiograph centering at the hinge point and extending the lines to the anterior cortex.
(4) Measure the horizontal distance (z, millimeters) on the anterior cortex, which is equal to the height of the wedge to be removed.

Measuring patellar height relative to the proximal tibia, that is, with the Caton-Deschamps (CD) index enables to predict to what extent the patella is heightened when conducting a supratuberosity ACWO. In this respect, a preexistent low patella can be altered toward normal (CD index ranges from 0.6 to 1.3). However, a preexistent patella alta should not be increased by the ACWO, and it is advised that after planning the correction, the patellar height, as calculated with the CD index, should not exceed the limits of patella alta (1.3) too much. In such cases, a transtuberosity or infratuberosity ACWO technique is advised.

An ACWO can also correct some coronal malalignment by resecting an asymmetric wedge, if the deformity lies in the tibial metaphysis. However, it is important to remember that correction of a coronal plane malalignment, especially tibial varus, must be prioritized if that deformity is greater than 5°.^50,51^ A medial opening wedge high tibial osteotomy does allow reduction of the PTS, especially if a patient-specific custom cutting guide is used to control this variable.^52^

## Authors' Preferred Technique of Anterior Closing Wedge Osteotomy

A slope-correction osteotomy at the level of tibial tuberosity, as described by Friedmann et al,^48^ but without a tibial tubercle osteotomy (TTO) is our preferred technique. This technique can preserve the native patellar height.^53^ The surgery can be done under neuraxial or general anesthesia. The patient is in a supine position with table flat, a thigh side support, and a foot stop at 90° knee flexion. A fluoroscopy unit is positioned on the opposite side to obtain lateral views of the tibia.

### Exposure

**Figure 6:**
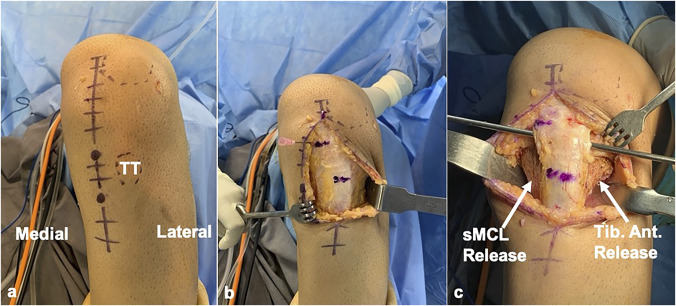
**A**, Photograph of the left knee showing surface marking for surgical exposure. **B**, Photograph showing the appearance after elevation of the fasciocutaneous flaps medially and laterally. **C**, Photograph showing the medial and lateral tissues released to expose the tibia and Hohmann retractors inserted. A Wissinger rod is passed posterior to the patella tendon at its insertion. sMCL = superficial medial collateral ligament, TT = tibial tuberosity, Tib. Ant. = tibialis anterior

An 8- to 10-cm. long paramedian incision just medial to the tibial tuberosity (TT) is made, such that 1/3 of the incision is superior and 2/3 inferior to the TT. Full-thickness fasciocutaneous flaps are elevated medially and laterally. The anteromedial tibia is exposed by releasing the anterior portion of the superficial medial collateral ligament, taking care to preserve its distal attachment and the posterior oblique ligament. The anterolateral tibia is exposed by elevating the tibialis anterior muscle off the bone with a Cobb elevator for at least 6 to 7 cm. distal to the TT. Hohmann retractors are placed on either side for exposure and for the protection of the dorsomedial and dorsolateral soft tissues (Figure 6).

### Osteotomy

**Figure 7:**
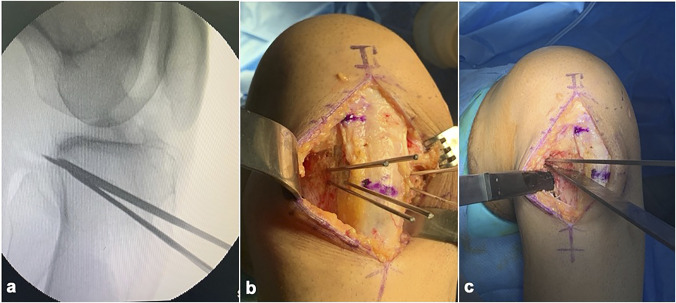
**A**, The Kirschner wires for marking the osteotomy wedge as seen on a lateral image-intensifier image. **B**, Clinical image of the superior and inferior Kirschner wires inserted from the anteromedial and anterolateral cortices. **C**, Photograph showing the osteotomes inserted on either side of the wedge.

**Figure 8:**
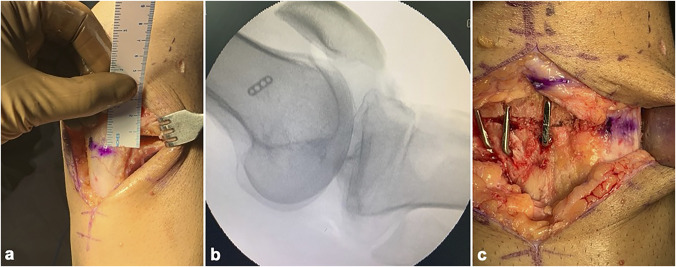
**A**, Photograph of the osteotomy as seen after removal of the bone wedge based anteriorly. **B**, The osteotomy is closed by extending the distal tibia as seen on the image-intensifier image. **C**, Photograph showing fixation of the osteotomy done with staples.

The osteotomy site is marked on the anteromedial cortex at the level of the TT with two 1.8-mm Kirschner wires under fluoroscopy control in the lateral view. These Kirschner wires are drilled toward the posterior cortex, aiming for the hinge point at the PCL tibial attachment site. Kirschner wires are drilled from the anterolateral cortex parallel to the previous wires under fluoroscopic control. The height of the wedge to be resected is measured and marked on the anterior cortex on both sides, distal to the Kirschner wires. Two 1.8-mm Kirschner wires are then drilled from these points to converge with the first two wires at the hinge point. An additional Kirschner wire of the same length as the previously inserted ones is then used to measure the intraosseous length of the inserted Kirschner wires toward the hinge point. The saw blade and osteotome used for the osteotomy are marked accordingly to prevent cutting the posterior cortex. The Kirschner wires are then cut short to allow working space for the saw. A thin saw blade (eg, 0.9 mm thick) is then used to resect the wedge of the bone under fluoroscopy, making sure to completely cut the anteromedial and anterolateral cortices while preventing cutting the posterior cortex and protecting the patella tendon. It is safer to stop the saw blade approximately 1 to 2 cm. short of the hinge. Osteotomes can be used to complete the osteotomy and remove the wedge of the bone carefully from behind the patella tendon (Figure 7). The wedge is then closed by gradually bringing the knee into extension by holding the distal 1/3 on the leg while gently applying some axial pressure on the leg and some anterior pressure at the osteotomy site with the opposite thumb to control the lever arm. The osteotomy must be closed gradually over several minutes and under fluoroscopy control (Figure 8, A and B).

### Fixation

Once the wedge is fully closed, provisional fixation can be done using two cross Kirschner wires with the knee in extension. The wires used to mark the osteotomy are then removed. The knee is positioned in approximately 60° flexion. The osteotomy is fixed using 1 to 2 staples (Biotek) each on the anteromedial and anterolateral cortices to achieve satisfactory stability. When inserting the staples, the hinge point can be protected by applying posterior support to the calf. These staples work according to the tension band principle with the intact posterior hinge. The knee is taken through a complete range of motion to assess stability of the osteotomy, which will determine the progression of rehabilitation (Figure 8, C).

### Revision Anterior Cruciate Ligament Reconstruction

The decision to conduct a concomitant revision ACLR or bone grafting of previous tunnels along with the ACWO has to be made prior. In such a case, graft harvest and preparation are done first. A bone–patella tendon–bone graft cannot be used if a TTO is being performed using the transtuberosity technique, and an alternative graft source must be planned for accordingly. Arthroscopy is performed to remove the torn ACL, clear the tunnels, and complete any additional meniscal or cartilage surgery. The new femoral tunnel is drilled next. An ACWO is conducted after this and completed till fixation. Finally, arthroscopy is then conducted to drill the tibial tunnel, pass the ACL graft, and fix it.

#### Rehabilitation

The knee is braced in full extension for 4 weeks to allow the osteotomy to heal and to prevent knee hyperextension. The hematoma caused by the osteotomy normally causes some scarring of the posterior capsular structures, therewith reducing the initial hyperextension found after the correction intraoperatively. Early passive range of motion can be allowed from 0° to 90° if one is confident of the osteotomy fixation. The initial goal is to reduce edema with cryotherapy and control pain by multimodality interventions. Immediate partial weight bearing with crutches can be allowed, unless this is precluded by additional meniscus or cartilage surgery. Radiographs at 4-week intervals to assess the osteotomy healing help determine the progression of the rehab program.

#### Complications of Anterior Closing Wedge Osteotomy

##### Hinge fracture

The hinge of ACWO lies in the area of the tibial PCL insertion posteriorly. A fracture of the hinge is possible if the saw or osteotomy violates the posterior cortex, if the osteotomy gap after removal of the wedge is closed rapidly without allowing for plastic deformation of the bone, or if the hinge is too thick, necessitating the application of excess force for closure. The difficulty of a hinge fracture lies in stabilizing the fracture because this area is not surgically accessible by an anterior approach and the relatively small proximal segment makes it difficult to control. Preventing reversal of the slope due to a pull of the PCL, rotational malalignment, and posterior displacement of the distal segment are essential. A direct stabilization of the hinge with a screw or staple is not easily possible. Fixation of the osteotomy with staples only would now result in an unstable construct because the tension band is lost. In such a situation, the osteotomy is preferably fixed with an angle-stable plate anteromedially and staples anterolaterally for additional stability, if needed. Extra stabilization with lag screws (anterolateral to posteromedial) may be done where feasible. Revision ACLR is preferably deferred to allow the unstable osteotomy to heal.

##### Patella alta

This is a theoretical possibility when a supratuberosity osteotomy is performed because of shortening of the infrapatellar length of the patellofemoral complex. It is a distinct disadvantage of this technique, and alternative methods which avoid this complication are hence preferred. This complication can be avoided by using transtuberosity or infratuberosity techniques (Table 2).

##### Implant related

Irritation from the staples or screws used for fixation of the ACWO is possible, especially on the anteromedial cortex. This usually necessitates removal of the offending implant after healing of the osteotomy.

##### Neurovascular injury

Because the osteotomy is conducted from anterior to posterior, injury to the popliteus neurovascular structures from Kirschner wires, saw, or osteotome is a distinct possibility. Conducting the osteotomy in 60 to 90° knee flexion, adherence to a meticulous technique including the intraosseous length measurement marking on saw blades and osteotomes, and conducting each step under fluoroscopy control can prevent this disastrous complication.

##### Knee hyperextension

The ACWO has the inadvertent effect of causing hyperextension of the knee. This can be problematic if the patient already had some hyperextension preoperatively, especially in an ACL-deficient knee. A method to anticipate the amount of hyperextension that will result after the correction has been described.^43^ It is important to keep a watch on the knee extension when closing the osteotomy and titrate the closure by stopping short of the planned correction if the hyperextension seems to be >10°. Then, part of the inserted wedge can be reinserted to fill the remaining gap. In the case of symptomatic hyperextension resulting from an ACWO, soft-tissue posteromedial advancement whether combined with posterior capsular shortening techniques can be tried to reduce symptoms.

## Results of Anterior Closing Wedge Osteotomy

There is limited literature available which reports on the clinical outcomes of an ACWO. Song et al^54^ published their results of slope-reducing osteotomies conducted along with primary ACLR. This philosophy is not widely accepted, and the ACWO is preferably reserved for patients with one or more failures of ACLR. The earliest results of this surgery were provided by Sonnery-Cottet et al^55^ in their retrospective series of five rerevision ACLRs combined with ACWO. The mean PTS reduced from 13.6° preoperatively to 9.2° after surgery while the mean differential anterior laxity reduced from 10.4 to 2.8 mm. Dejour also reported a retrospective series of nine patients who underwent a second revision ACLR and ACWO with a median follow-up of 3.6 years.^56^ The mean PTS decreased from 13.2° ± 2.6° to 4.4° ± 2.3° after surgery. There was notable improvement in Lysholm and IKDC scores with no additional ACL graft retears. Both Sonnery-Cottet et al and Dejour et al conducted transtuberosity osteotomies without a TTO.^55,56^ More recently, Akoto et al^57^ reported their retrospective series of two-stage surgeries, ACWO followed by revision ACLR, in 21 patients. All the patients also had high-grade pivot shifts and underwent a lateral extra-articular tenodesis during the second stage. The mean PTS reduced from 15.3° to 8.9°, and the mean side-to-side difference of anterior laxity reduced from 7.2 to 1.1 mm. There were no failures of the revision ACLRs, and no patient had a positive pivot shift at the final follow-up. The postoperative functional scores (Lysholm and Knee Injury and Osteoarthritis Outcome Score) were good to excellent for all patients.^57^ Nishino et al^58^ published a case report of an ACWO and ACLR conducted for salvage of a previous opening wedge HTO, which had led to symptomatic anterior knee instability due to an inadvertent slope increase. Thus, the limited data available do demonstrate the effect of an ACWO in preventing failure of revision ACLRs when the PTS is exaggerated.

## Summary

A high PTS is a well-known risk factor causing failure of ACLRs. An ACWO may be contemplated when the lateral tibial slope is greater than 12°. Careful surgical planning and meticulous execution are required to conduct this complex surgery and avoid complications.
